# Towards a Secure and Scalable Maritime Monitoring System Using Blockchain and Low-Cost IoT Technology †

**DOI:** 10.3390/s22134895

**Published:** 2022-06-29

**Authors:** Warlley Paulo Freire, Wilson S. Melo, Vinicius D. do Nascimento, Paulo R. M. Nascimento, Alan Oliveira de Sá

**Affiliations:** 1 Adm. Wandenkolk Instruction Center, Brazilian Navy, Rio de Janeiro 20180-003, RJ, Brazil; paulo.freire@marinha.mil.br; 2 National Institute of Metrology, Quality, and Technology, Duque de Caxias 25250-020, RJ, Brazil; wsjunior@inmetro.gov.br (W.S.M.J.); prnascimento@inmetro.gov.br (P.R.M.N.); 3 CISMAR, Brazilian Navy, and COPPE/Sistemas-UFRJ, Rio de Janeiro 21941-972, RJ, Brazil; dalto@cos.ufrj.br; 4 LASIGE, Departamento de Informática, Faculdade de Ciências, Universidade de Lisboa, 1749-016 Lisboa, Portugal

**Keywords:** permissioned blockchain, maritime monitoring system, hyperledger fabric, automatic identification system, Docker

## Abstract

Maritime Domain Awareness (MDA) is a strategic field of study that seeks to provide a coastal country with an effective monitoring of its maritime resources and its Exclusive Economic Zone (EEZ). In this scope, a Maritime Monitoring System (MMS) aims to leverage active surveillance of military and non-military activities at sea using sensing devices such as radars, optronics, automatic Identification Systems (AISs), and IoT, among others. However, deploying a nation-scale MMS imposes great challenges regarding the scalability and cybersecurity of this heterogeneous system. Aiming to address these challenges, this work explores the use of blockchain to leverage MMS cybersecurity and to ensure the integrity, authenticity, and availability of relevant navigation data. We propose a prototype built on a permissioned blockchain solution using HyperLedger Fabric—a robust, modular, and efficient open-source blockchain platform. We evaluate this solution’s performance through a practical experiment where the prototype receives sensing data from a Software-Defined-Radio (SDR)-based low-cost AIS receiver built with a Raspberry Pi. In order to reduce scalability attrition, we developed a dockerized blockchain client easily deployed on a large scale. Furthermore, we determined, through extensive experimentation, the client optimal hardware configuration, also aiming to reduce implementation and maintenance costs. The performance results provide a quantitative analysis of the blockchain technology overhead and its impact in terms of Quality of Service (QoS), demonstrating the feasibility and effectiveness of our solution in the scope of an MMS using AIS data.

## 1. Introduction

With the exponential increase in cyberattacks and their impacts [1,2,3,4], nations and organizations worldwide seek solutions to defend their cyber domains. In this scenario, the maritime community also pursues ways to secure its digital systems, which tend to have their attack surface increased by the adoption of new technologies. Like many sectors of society, the maritime sector is duly benefiting from cutting-edge Information Technologies (IT) and Operational Technologies (OT). Ships are becoming complex cyber–physical systems that integrate navigation sensors, information systems, and control systems. Digital technologies are increasingly being used in Maritime Monitoring Systems (MMSs) [5] to collect and fuse relevant navigation information, such as the voyage data transmitted through ships’ Automatic Identification Systems (AISs).

However, at the same time that digital technologies bring benefits to the maritime sector, they also expose it to threats typical of the cyber domain. Not by chance, the International Maritime Organization (IMO) published the Guidelines on Maritime Cyber Risk Management [6], which encouraged a global movement to strengthen cybersecurity in the maritime environment. Recently, in the same direction, the United States of America launched its National Maritime Cybersecurity Plan [7] to address the potentially catastrophic risks to national security and economic prosperity caused by the maritime transport system’s increasing dependence on IT and OT. Data provided in [8], for instance, show an increase of 900% in maritime cyberattacks in the last three years, evidencing the need for security tools that can help to mitigate cyber-electronic vulnerabilities [9].

In this challenging scenario, blockchain-based systems can play an important role, leveraging data security through decentralization and sophisticated encryption mechanisms. Blockchain technology, which has become mainstream in the financial sector in the last decade due to its wide proven capabilities, now is spreading its application to the military and other sectors, helping to leverage security in critical systems [10]. Due to the growing use of the Internet of Things (IoT) in the maritime environment, with applications in autonomous shipping, navigation, monitoring, and other critical applications, the need for a secure and reliable communication system is bold [11]. Note that, according to the e-navigation Strategy Implementation Plan [12], which aims at a large-scale maritime information system, improved reliability and data integrity are among the shipboard user needs and priorities.

This work focuses on the vulnerabilities of a national-scale MMS, where the sensing devices are isolated and often in dangerous environments. In this scenario, attackers could try to tamper with physical sensors or even modify data from Radio Frequency (RF) communication channels, compromising data integrity and authenticity [11]. In an even worst scenario, they could use tactics similar to the ones in the SolarWinds hacking [1] to access the MMS database and put the system availability at risk.

To overcome these risks, we propose a blockchain-based MMS that could secure the integrity, authenticity, and availability of sensing data from vessel traffic services and relevant for life’s safety at sea. In the proposed solution, the Raft consensus algorithm is used to provide fault tolerance, and permissioned blockchain mechanisms restrain the access of entities through cryptography mechanisms, being more suitable for an MMS. We evaluate how this solution meets Quality of Service (QoS) requirements in terms of the time response and management of large amounts of data. Additionally, we assess the feasibility and performance of the proposed solution when blockchain clients are implemented on a low-cost IoT device, with limited computational resources, taking into account different hardware configurations. To allow performance evaluation with different hardware profiles, this work proposes a methodology where blockchain clients responsible for transmitting AIS data are dockerized on a Raspberry Pi.

The main contribution of this paper is four-fold:

- Develop a functional permissioned blockchain MMS prototype that receives and securely stores AIS sensing data. The prototype is implemented on the Fabric platform and endowed with the Raft consensus algorithm for fault tolerance;
- Integrate the prototype with a low-cost AIS receiver, developed by the Brazilian Navy, demonstrating the feasibility of a blockchain-based MMS in a real environment where the blockchain client runs on devices with limited computational resources;
- Evaluate the system performance on different hardware configurations for the blockchain client, using a methodology where the blockchain-based low-cost AIS receiver is dockerized on a Raspberry Pi;
- Analyze, through experiments, the system’s overall performance, and quantitatively determine the blockchain technology’s overhead, evaluating if its performance meets desirable QoS levels.

The remainder of this paper is organized as follows: Section 2 presents the related works; Section 3 describes an MMS, characterizes the attack models that can threaten an MMS, and presents the concept of a blockchain-based MMS; Section 4 explains the methodology used to validate the proposed solution and assess its performance with different client hardware configurations; Section 5 presents the results obtained through experiments with a prototype system; finally, Section 6 gives the conclusions and directions for future works.

## 2. Related Work

In this section, we explore available articles related to maritime systems’ cybersecurity, IoT sensing systems, and blockchain technology to provide the background of our research. Initially, Section 2.1 presents important knowledge about the blockchain technology, its main mechanisms and implementation, and how it achieves consensus in a distributed network. In Section 2.2, we analyze relevant works leveraging the blockchain technology in the IoT and sensor networks. Section 2.3 presents works evaluating maritime systems regarding cybersecurity and guidelines to address cyber risks in these systems. Finally, Section 2.4 shows works applying the blockchain technology to improve maritime systems.

### 2.1. Blockchains in a Nutshell

Blockchain is an emerging and disruptive technology that poses a solution to provide trust among parts that do not trust each other [13,14,15]. The blockchain’s core consists of a distributed, decentralized append-only data structure designated as a ledger [16]. A peer-to-peer network of independent peers is necessary to implement a blockchain. The peers store data in cryptographically chained blocks and replicate those blocks among themselves, ensuring that each participant in the network has its consistent local ledger copy. A complete blockchain platform supports data and also metadata storage, which means that data can yield self-executable workflows called smart contracts [16]. Cryptography directives and consensus protocols in a blockchain ensure data integrity and consistency [14].

Consensus protocols are one of the more critical aspects of blockchains’ performance currently [15]. The literature usually presents consensus as two main groups: proof-based and vote-based [16]. Proof-based consensus (e.g., PoW, PoS, PoET) usually works like a lottery and enables any peer to participate without prior authorization [15]. In contrast, vote-based consensus (e.g., Byzantine fault-tolerant, crash fault-tolerant) depends on a quorum of identified peers, resulting in permissioned blockchains [14]. In practical aspects, proof-based consensus enables many more participants and incentivizes mining mechanisms, making it easier to attract peers to maintain the blockchain infrastructure. Most blockchain-based cryptocurrency applications rely on this network and protocol, motivating peers to keep the ledger consistent in exchange for financial profits. As a drawback, proof-based consensus tends to present poor performance in blocks’ and transactions’ throughput. On the other hand, vote-based consensus (and consequently, permissioned blockchains) cannot count on free incentive mechanisms. They depend on a previous agreement among the consensus quorum members, which usually considers that maintaining the blockchain is beneficial for all involved parties. Consequently, vote-based consensus aggregates better performance and higher throughput rates, even a hundred-times faster than proof-based consensus [14,17].

Adopting a blockchain platform is usually the more practical way to implement blockchain-based solutions. Blockchain platforms work similarly to a distributed operating system, providing elementary features that accelerate new applications’ conception [16,18]. At the moment, some blockchain platforms have successfully consolidated themselves as a reference implementation. A well-known platform is Bitcoin [13], which is potentially the first blockchain platform proposed to work as a payment network and which became remarkable due to its cryptocurrency popularity. Another important blockchain platform is Ethereum [19], which supports smart contracts’ implementation in the Solidity language. Both mentioned platforms use proof-based consensus, becoming permissionless platforms. A third example is the Hyperledger Fabric [18], described as a modular platform with high customization flexibility. Although Fabric implements consensus as a pluggable module, it usually constitutes a permissioned blockchain platform. Since 2018, Fabric has included the Raft [14,15,16] consensus natively. Raft is a Crash Fault-Tolerant (CFT) consensus protocol based on the quorum leader’s concept [14], which coordinates the member votes and yields new blocks. Many authors describe Raft as a highly efficient consensus algorithm, being propagated and employed in several distributed system implementations [17]. Besides, Raft is easier to understand and more convenient to implement. Raft supports crash fault since 50% plus one of its quorum is functional.

Blockchain technology has been widely debated by many authors and has proved its capabilities beyond its primary use in cryptocurrency. However, this technology also imposes some challenges of scalability, especially if used within large IoT networks. Seeking to address the scalability challenge while securing critical data security requisites, we analyzed works that presented important insights and frameworks in this scope.

### 2.2. Blockchain in IoT and Sensor Networks

Different works discuss blockchain technology applications in monitoring systems. Al-Sahan et al. [20] presented a permissioned blockchain implementation on Fabric to build a public national surveillance system, integrating a heterogeneous array of entities composed of public and private stakeholders. This system combines blockchain decentralized properties with machine learning algorithms designed for facial recognition, allowing the system to precisely identify people through a widely distributed surveillance system. The authors emphasized Fabric’s modular framework as a key feature to effectively integrate different surveillance subsystems. They also demonstrated that Fabric deals with large amounts of data, delivering a good overall performance.

Melo et al. [21] presented a comprehensive framework that describes how to implement a blockchain-based system to monitor and protect critical infrastructures, securing data availability and integrity. The authors compared the performance of two distinct blockchain implementations using the Ethereum and Fabric platforms. Their analysis showed Fabric’s slight advantage in terms of throughput, modular framework, and better development tools. In turn, Ethereum presents a simpler configuration and well-predictable costs.

To address the scalability challenge of blockchain-based systems, Bandara et al. [22] introduced a lightweight blockchain for IoT. In their work, the authors also used the Apache Kafka consensus to enhance scalability and real-time transaction execution on the blockchain. The work also made a contribution by reducing performance overheads, by using sharding-based data replication. However, the work contributions lack methods that could help prepare IoT sensors, a big challenge to system scalability. Gil et al. [23], on the other hand, pursued this gap, using Docker containers to compact together all the dependencies and libraries used by a blockchain client. The work goal is to reduce the time spent on setting up a blockchain, thus contributing to easing the system scalability challenges.

Furthermore, Honar et al. [24] also addressed the scalability problem of IoT blockchain-based systems, presenting an implementation on the HyperLedger Fabric platform and analyzing the performance of the network compounded by resource-constrained IoT devices, as in an MMS. The authors tried to evaluate the processing power and storage issues through a performance analysis of their practical implementation. Their results showed that transaction throughput and latency, resource consumption, and network use can be optimized by their HyperLedger Fabric implementation while security requirements are achieved, thus being suitable in many IoT scenarios.

### 2.3. Cybersecurity of Maritime Systems

In the current maritime landscape, ship identification systems (e.g., AIS), sensors (e.g., radar systems), and integrated maritime monitoring systems are essential for navigation safety and safeguarding of human life at sea [25]. These marine-specific digital technologies are increasingly integrated with common IT, forming complex computer-based environments prone to cyberattacks [9], which motivates the rise of regulations/policies [6,7] and research efforts to mitigate threats. In this context, related works focusing on the cybersecurity of maritime monitoring systems are discussed hereafter.

Aligned with the IMO guidelines [6], the study presented in [26] introduces a method for assessing cyber risks in ship navigation systems, encompassing network penetration tests, detection and analysis of vulnerabilities, and specific analyses for critical onboard assets. Similarly, in [27], the authors presented a study on the cybersecurity of a shipboard Integrated Navigation System (INS), which combines Electronic Chart Display Information Systems (ECDISs), radar/ARPA systems, AISs, and other ship sensors to allow centralized access to navigation information. The authors applied a vulnerability scanner to examine the ship’s security and revealed risks deriving from the weaknesses of the INS operating system, mostly related to the possibility of remote code execution and unauthorized access gaining.

In Kavallieratos et al. [28], the authors addressed the security requirements of a cyber-enabled ship, applying the Secure Tropos [29] development methodology to systematically elicit the security requirements of the three most vulnerable ship subsystems, according to them: the ECDIS; the Global Maritime Distress and Safety System (GMDSS); and the AIS. From the perspective of the onboard AIS, the authors highlighted the need for security mechanisms to (a) prevent the loss of AIS information; (b) protect voyage-related data against tampering or damage; and (c) use authentication mechanisms to uniquely identify the actors reading, modifying, and transmitting AIS data.

Androjna et al. [30] identified the AIS vulnerabilities and provided a case study of a spoofing attack event that occurred near Elba in December 2019. In this case, thousands of fake AIS streams were artificially generated with different identification codes, positions, routes, and speeds, making clear that the typical AIS can be easily spoofed with fake information.

To improve the security of AISs, Goudossis et al. [31] proposed the use of symmetric cryptography and public-identity-based cryptography. The solution is designed to be used within single-hop broadcasts among ships at sea, in AIS ad hoc networks, and can benefit integrated systems such as e-navigation [5]. Kessler [32] presented another research work aimed at protecting AIS data with security mechanisms. It introduces the protected AIS (pAIS), which uses public key cryptography to provide authentication and message integrity and, thus, mitigate AIS security weaknesses. Note that all these works focused on the cybersecurity of on-board naval systems and systems for exchanging navigation information between ships, the security of the MMS that integrates these data on a large scale, like the one aimed at the present work, being little explored.

### 2.4. Blockchain in Maritime Systems

Rahimi et al. [11] developed a solution using an Ethereum blockchain to secure data in an MMS. Their system uses buoys and Unmanned Aerial Vehicles (UAVs) to gather maritime sensing data and send them to a data fusion center on shore through a mesh topology network. The authors suggested a proof of authority consensus protocol, more energy-efficient and tailored to a permissioned blockchain. Through MATLAB simulations, the authors evaluate the performance of the system in terms of delay and throughput. The blockchains resulted in an overhead of 13∼18% in delay and 12∼16% in throughput, which are acceptable QoS levels.

Additionally, Zhang et al. [33] explored the non-tampering and non-forgery characteristics of the blockchain technology to deliver a system capable of integrating many heterogeneous and modular IoT devices involved in a Maritime Transportation System (MTS). The authors emphasized the requisite of a shared and controlled access mechanism that must not be manipulated or accessed by unauthorized parties in order to achieve data integrity and authenticity in an MTS. The proposition was validated through experimentation, and the results showed a security improvement of 8% and a transaction processing speed improvement of 6% while resisting attacks such as replay attacks and camouflage attacks. In the same scope, Jiang et al. [34] presented a lightweight blockchain, tailored for edge IoT-enabled MTS and capable of guaranteeing the security of sensor data. This work explored the low-energy-consumption characteristics of the proof of stake consensus, reducing the energy used by IoT devices. Their results showed a consumption reduced by 78% compared with traditional proof of stake blockchain systems, being an important achievement in order to be applied to IoT sensing devices in remote platforms, such as drones and buoys, as discussed in our work.

Despite the solid effort in developing functional implementations of blockchain technology to secure data in monitoring systems, to the best of our knowledge, no works present an implementation of a Fabric-permissioned blockchain in MMSs. This work pursues fulfilling this gap, developing a practical blockchain implementation using the Fabric platform and evaluating its performance in a real maritime monitoring scenario where the blockchain clients run on low-cost devices with limited computational resources.

## 3. System Model

This section describes the characteristics, topology, and workflow of a maritime monitoring system. First, Section 3.1 presents a generic MMS, composed of a sensing IoT network that stores its data in a conventional database (SQL). Then, Section 3.2 presents an attack model for this conventional MMS, using known and documented vulnerabilities. Section 3.3 presents the concept of a blockchain-based MMS, detailing its operation characteristics. It is explained how the blockchain ensures the integrity and authenticity of sensing data through asymmetric cryptography and how it achieves consensus within the peers that compose a wide-scale MMS. Finally, Section 3.4 presents a security analysis that discusses the resilience of a conventional and a blockchain-based MMS against the attacks presented in Section 3.2. It is important to stress that this analysis is not limited to an MMS, but can be applied to any sensing IoT network using conventional databases.

### 3.1. Maritime Monitoring System

Maritime Domain Awareness (MDA) [35,36,37] is an area of study to understand events, activities, and military and non-military circumstances within a maritime environment using available data sources (sensors mainly). At sea, mariners must be situationally aware to conduct operations effectively. Fundamentally, the main challenge is related to the detection of obstacles and the prediction of close-range encounter situations. As such, effective collision avoidance can be seen as a key component of safe shipping systems.

In this context, the Maritime Authorities have a great challenge to monitor their maritime domain and ensure the use of their Exclusive Economic Zone (EEZ). Activities such as illegal fishing, illicit traffic, water pollution, and even piracy need to be tackled daily. The AIS is the utmost tool used in maritime monitoring and one of the most common sensors utilized in MMSs. Through AIS receivers, the MMS collects ships’ data in wide monitored areas and shows an organized view to users. Furthermore, machine learning models can be used on AIS data to predict vessels’ behavior. In addition to AISs, an MMS can also use data from other sensors such as radars, cameras, etc. Note that, for effective monitoring, the sensing devices of an MMS may need to be installed in buoys and UAVs, with limited computational and energy resources. Additionally, there is a scalability problem in deploying such a system, with the need to prepare hundreds of heterogeneous sensors to be able to communicate wirelessly throughout an enormous area.

Currently, according to the Safety of Life at Sea (SOLAS), issued by IMO and adopted by the Maritime Authorities, ships with more than 300 gross tonnages worldwide are required to carry an AIS transponder on board. The AIS is an onboard vessel-tracking system that allows vessels to report their positions periodically. For monitoring the vessels, AIS stations need to be installed in strategic places distributed on their coast to receive AIS signals from ships sailing within a radius of approximately 40 MN. The ships provide AIS data such as MMSI, name, latitude, longitude, speed, and direction. The AIS stations can be composed of antennas, frequency demodulators, and low-cost Linux-based devices, such as a Raspberry Pi. The AIS stations can be installed in remote places where there are no electricity and communication infrastructures, such as buoys and UAVs. To circumvent these constraints, AIS receivers can be used coupled with batteries and solar panels, having low energy consumption as a requirement for operation in these places.

It is worth mentioning that, to cover a wide maritime area, a substantial number of sensors must be deployed, imposing a great scalability challenge. The hardware of these devices must be able to process the data acquired by the sensors and prepare them for transmission. Each device must be able to communicate through a WMAN in a mesh topology, as suggested by Al-Saadi et al. [38], being capable of operating as a relay to transmit the sensing data of nearby sensors. These data are typically received by a data fusion center covering a specific maritime area under the responsibility of a Naval District (ND). Then, each ND aggregates the sensing data of its area and transmits them to be stored by the Naval Authority. The MMS can store the sensing data in two distinct ways: in a centralized database; or in a distributed redundant database.

Another factor that must be taken into account is the security of the sensing data. The sensors are installed in remote places; hence, they can be susceptible to some types of attacks and have their data changed. To emphasize the relevance of the area, Androjna et al. [30] presented the importance of cybersecurity in the maritime environment, highlighting the main threats and vulnerabilities of the *Global Navigation Satellite System* (GNSS), ECDIS, and AIS that affect maritime safety. The article recommends that the maritime community implement a robust cybersecurity system and use encrypted signals to protect against spoofing and other maritime cyber threats.

Considering active monitoring of the entire coast of a country, ensuring the security of an MMS is undoubtedly a major challenge. Aiming to evaluate the vulnerabilities of the aforementioned system, in Section 3.2, we analyze possible attacks against the integrity, authenticity, and availability of the sensing data. After, in Section 3.3 and Section 3.4, we propose our blockchain-based MMS and evaluate how it can mitigate these attacks, respectively.

### 3.2. Attack Model

Our attack model embraces four different scenarios with known vulnerabilities (e.g., PostgreSQL exploits) as attack vectors and some more sophisticated attack techniques, such the supply-chain attacks on SolarWinds [1]. The four attack scenarios with their respective attacker’s capabilities/restrictions are:

1. **Availability attack**: Attackers try to disable an MMS using a supply-chain attack as an initial attack vector. The attacker has the following capabilities/restrictions:
  - Infiltrate the Naval Authority’s private network using a backdoor opened by a supply-chain attack;
  - Explore known database vulnerabilities (e.g., malware injection on PostresSQL—CVE-2019-9193 [39]);
  - Impossibility to attack all NDs due to the complexity and the need to successfully exploit multiple attack vectors.
2. **Integrity attack**: Attackers try to tamper with stored sensing data in an MMS. The attacker steals or coerces an internal agent to obtain his/her credentials. With the credentials, attackers could access the Naval Authority’s private network and gather network settings’ information to fulfill the database attack, compromising integrity. The attacker has the following capabilities/restrictions:
  - Access Naval Authority’s private network with legitimate credentials;
  - Modify and corrupt data, while remaining covert;
  - Impossibility to attack all NDs due to the complexity and the need to successfully exploit multiple attack vectors.
3. **Collusion Attack**: Corrupted servers can cooperate in disabling the entire system, partially or totally degrading the system’s routing and networking capabilities, compromising data integrity and availability [40]. The attacker has the following capabilities/restrictions:
  - Capability to compromise more than one of the system servers in different NDs;
  - Modify and corrupt data in compromised servers;
  - Compromised servers can communicate with others;
  - Impossibility to corrupt all system servers.
4. **UAV hijacking**: Attackers try to spoof UAV–operator communications to gain control of the UAV or to remove it from its operational area. They explore the lack of authentication in UAV–operator RF communications. The attacker has the following capabilities/restrictions:
  - Eavesdropping on UAV–operator communications;
  - Spoofing UAV control messages;
  - The attacker has to be in the RF UAV–operator coverage area.

### 3.3. Blockchain-Based MMS

Aiming to leverage sensing data security, we propose a blockchain-based MMS implemented in the Fabric platform that ensures data integrity, authenticity, and availability. We also address a practical MMS scalability problem regarding configuring sensing devices on a large scale, using Docker containers to compact together all the dependencies and libraries required for the blockchain client operating in the sensing devices. Furthermore, we seek to optimize the resource consumption in these devices, limiting the container CPU and memory usage, thus requiring less computational power and energy to operate.

**Figure 1:**
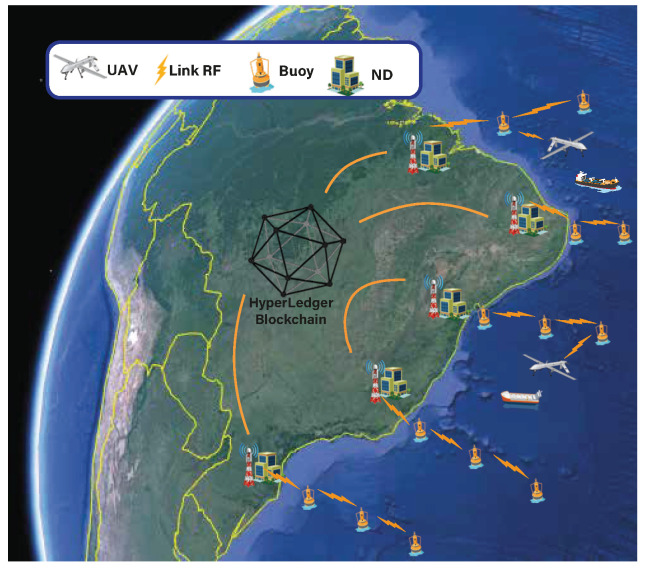
Blockchain-based MMS.

In our system, buoys and UAVs behave as blockchain clients, possessing their respective cryptographic credentials. Fabric’s Membership Service Provider (MSP) manages the keys and characterizes the blockchain’s permission nature. The clients are equipped with a low-cost AIS receiver developed by the Brazilian Navy, composed of a VHF whip antenna, a Software-Defined Radio (SDR) frequency demodulating dongle, and a Raspberry Pi 3. Figure 1 illustrates the blockchain-based MMS topology.

The blockchain implementation uses Fabric’s standard configuration [18] tailored to be integrated into an MMS. Clients transmit sensing data in transactions to the blockchain peers in the ND responsible for the monitoring area. The client application consists of Python 3 modules able to request smart contract functions using the Fabric Python Software Development Kit (SDK) [41]. The Fabric SDK delivers an abstraction layer, which allows easy application development and interacts with the blockchain in many different manners [42].

The client application processes the NMEA (NMEA: communication data format standardized by National Marine Electronics Association and used by GPS, AIS, Vessel Traffic System (VTS), and other applications) data received by the VHF whip antenna and analog–digital conversion by the SDR dongle. It accesses NMEA data directly on the SDR control application port or through a text file containing NMEA entries. Each NMEA string is sent, as a blockchain transaction, by the client to an endorsing peer in the respective ND and processed by the smart contract.

**Figure 2:**
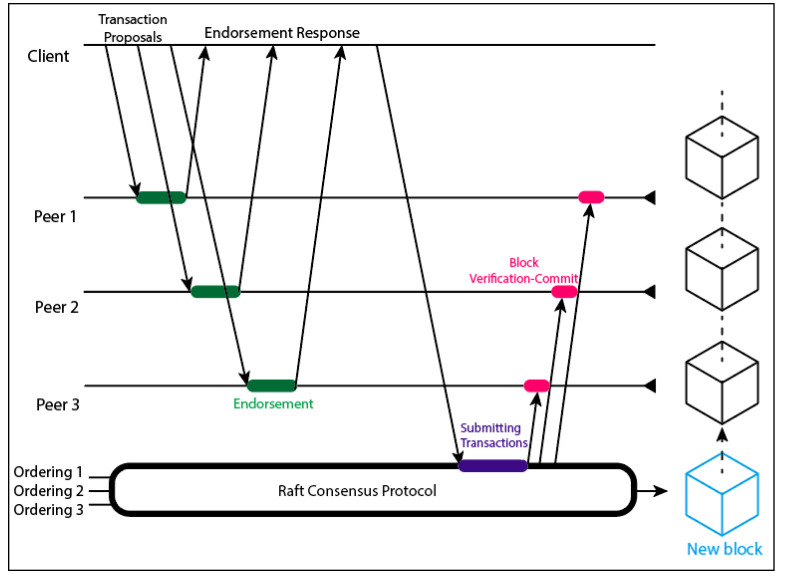
Raft consensus workflow [22].

The endorsing peers are responsible for processing each transaction and verifying data authenticity and integrity. The transaction invokes a specific smart contract function that can query the ledger, send a blockchain node command, or process the sensing data before storing them in the ledger. The endorsing peers sign the transaction response with their private keys and return it to the invoker. In turn, the client collects endorsements until it has the number of endorsements established in the system policy. This number is easily adjustable to meet different security levels, although this demands more processing power due to transactions being signed with asymmetric cryptography. The client then sends the endorsed transaction to the orderer service. Figure 2 illustrates this workflow.

**Figure 3:**
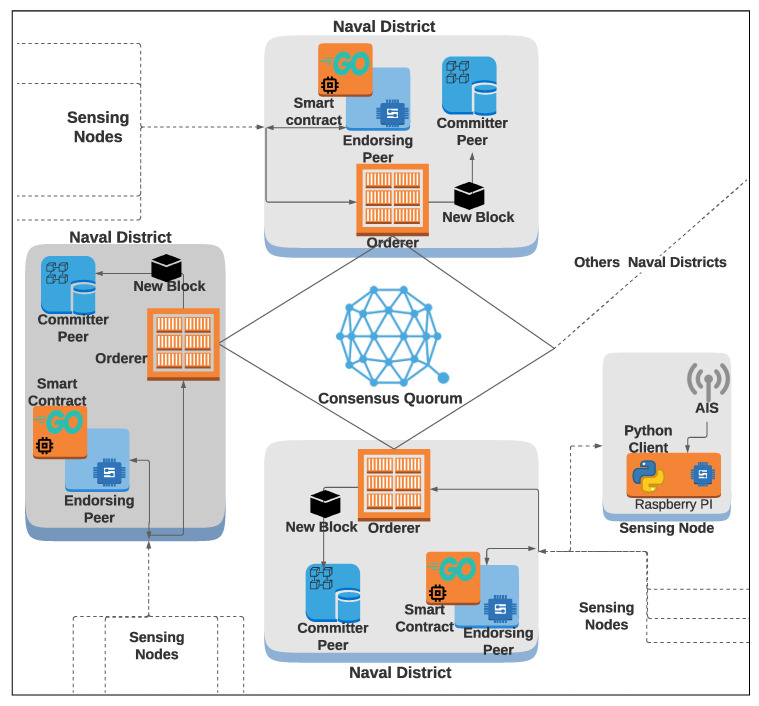
System architecture.

The orderer service is responsible for receiving all signed transactions and establishing the new block’s final order. In our deployment, we configured the orderer service to use Raft consensus, aiming to explore its advantages in terms of simplicity, ease of implementation, and crash fault tolerance, as discussed in Section 2.1. The orderer service broadcasts the new block to all blockchain nodes. Finally, committer peers verify the block and irreversibly add it to the ledger. Figure 3 shows the system architecture and the aforementioned blockchain entities’ arrangement. Transactions that present any inconsistency are not added to the ledger and are stored for further auditions. All data in the ledger become immutable, and no mechanism can modify them.

### 3.4. Security Analysis

**Figure 4:**
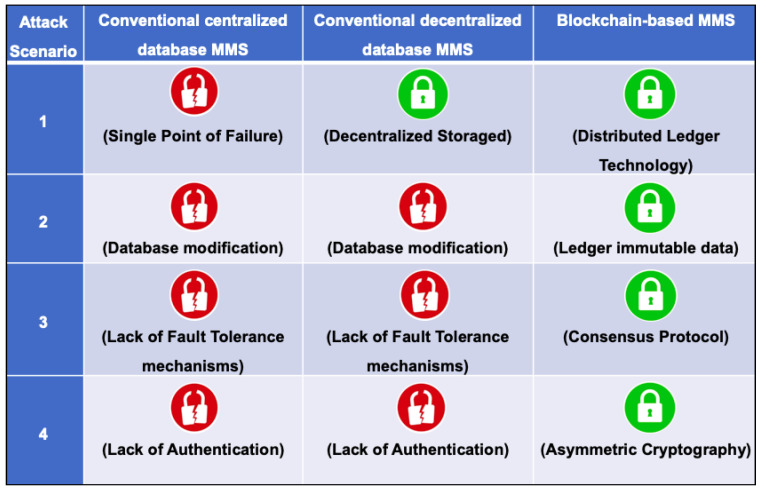
Security analysis.

This section presents a security analysis comparing a conventional MMS (with centralized and decentralized databases) and a blockchain-based MMS regarding the attack model described in Section 3.2. Figure 4 summarizes the analysis result, labeling with a red lock if the system is vulnerable to the attack and with a green lock if the system is resilient to the attack, also presenting the main reason for the success or failure of the system security in each scenario.

Firstly, we analyze the conventional centralized database MMS. It is vulnerable to the first attack scenario due to the storage of sensing data in only one server, resulting in a Single Point of Failure (SPF). It is vulnerable to the second scenario due to the database server accepting modifications to stored data. It is also vulnerable to the third scenario due to its lack of fault tolerance mechanisms. Finally, it is vulnerable to the last scenario due to its lack of authentication on UAV–operator communications.

The decentralized database MMS differs from the centralized one just in the first attack scenario due to its distributed data storage. If an attacker successfully breaks into a single ND, data would be safe on the other servers. However, in the other three scenarios, the decentralized MMS would perform like the centralized one.

The blockchain-based MMS is resilient against all four attack scenarios. It resists the first scenario attack due to its distributed ledger technology storing the sensing data in different nodes, unsettling the SPF. It resists the second scenario because all data added to the ledger become immutable and the attacker can no longer modify the stored data. It resists the collusion attack if the attacker corrupts less than 1/3 or 1/2 (depending on the consensus protocol) of all blockchain nodes due to the fault tolerance mechanism of the consensus protocol. Finally, it also resists the last attack scenario, securing UAV–operator communications through asymmetric cryptography and the MSP key management.

## 4. Methodology for Performance Assessment

For an MMS to be able to cover large areas, it is essential to launch numerous equipment in distinctive spots to collect data. Hence, it is vital to have a methodology to discover the minimum resources necessary for a device to run a blockchain client application. Otherwise, it would be adverse to buy several devices and test each one to decide which presents the best value for the money. This situation is undesirable for some reasons. First, we want to avoid the cost of buying many devices and testing all of them. Second, as not all IoT devices are programmed using just one technology, we would need to develop the same application in more languages for the experiments. Therefore, it was necessary to develop a methodology to simulate different devices with different memory and CPU parameters that handle processing our MMS blockchain-based client applications.

One solution to this problem is to build a Docker container image with all the necessary software pre-compiled and instantiate containers, varying the resource parameters. When using Docker to instantiate a container, one can set how many resources the host will share with the container. According to Docker documentation, the host kernel handles the container processes. However, it separates them from host processes using the operational system namespace and group configurations. This way, one can control how much CPU and memory the container’s tasks can consume. By default, the container access to CPU cycles is unlimited. Still, a user can set constraints by configuring the Linux CFS Scheduler [43] or using the real-time scheduler. Docker does not encourage setting up a container to use the real-time scheduler since it is an advanced kernel-level feature, and incorrect values can cause your host system to become unstable or unusable.

Recent studies used containers as a distributed processing of simulations and improved simulations with a large number of devices in the same network. Hedström and Gudjonsson [44] presented a use case of using the AWS IoT Device Simulator service to simulate a fleet of drones. Under the many service layers, AWS uses containers to instantiate the IoT devices and the backends with which the devices should communicate. Kirchhof et al. [45] proposed a service architecture to simulate connected vehicles in a realistic environment. In this scenario, the hardware requirements explode as the number of automobiles grows and the simulated area becomes large. Therefore, based on their experience in the agile software engineering process and simulation technology, they proposed a simulation-as-a-service approach that uses containers to help scale the solution and abstract the processing distribution complexity. Shahin et al. [46] presented a systematic review of works with different strategies and architectures for Modeling and Simulation-as-a-Service (MSaaS). We can note, by reading the articles, that many approaches used a container solution to provide a distributed, parallel, and scalable solution together with cloud services.

Although those works help validate the idea of using containers to simulate IoT devices, they do not describe in-depth how to set the devices’ parameters during the configuration phase of the simulation. This lack of information brings the opportunity to explore ways to cope with this problem. Our approach uses a Docker-compose file to set the resource configuration, which allows setting the limits of the CPU and memory to be consumed by a container. The image preparation is divided into two steps:

- **The first step** is to build a Docker image with all the client application dependencies pre-compiled. The Dockerfile composed for this work uses a base image from the Python repository on Docker Hub. This image has Python version 3.7 running on a Debian Buster operational system compiled for the ARM architecture. The Raspberry Pi architecture and previous experience using the Hyper Ledger Fabric SDK for Python were the motivation to help determine the image base.
- **The second step** is to compile the Hyper Ledger Fabric SDK and its dependencies.

Therefore, the main objective of the Docker image built for this experiment is to have a working environment with the Hyper Ledger Fabric SDK pre-compiled, which could provide a fast and scalable start for use in different devices. The image built can be found on Docker Hub [47] with a README that explains how to use it. The README has an example of the Docker-compose.yaml that we coded to manage the container building. The key “deploy” is where we set the resource limits. Note that the key “build” points to the same directory where Docker-compose.yaml is. Therefore, the Docker-compose application will look for a Dockerfile alongside the Docker-compose file to know how to build the container and what application to run. The README also has an example of a Dockerfile to help users with the container setup process.

We set several CPU and memory boundaries during the experiments to determine the minimum configuration necessary to run the MMS blockchain client application on a low-cost IoT device. Furthermore, we investigated situations where adding more resources to the client device does not bring substantial benefits to the application’s runtime. It is important to note that Docker-compose brings the possibility to rapidly instantiate many containers with different setups just by describing them on the Docker-compose.yaml script.

## 5. Experiment and Results

In this section, we quantitatively analyze the processing overhead caused by the blockchain technology through an experiment integrating our blockchain prototype with the Brazilian Navy’s low-cost AIS. We also evaluate the performance of the MMS blockchain-based client using different hardware configurations.

### 5.1. Experiment Description

**Figure 5:**
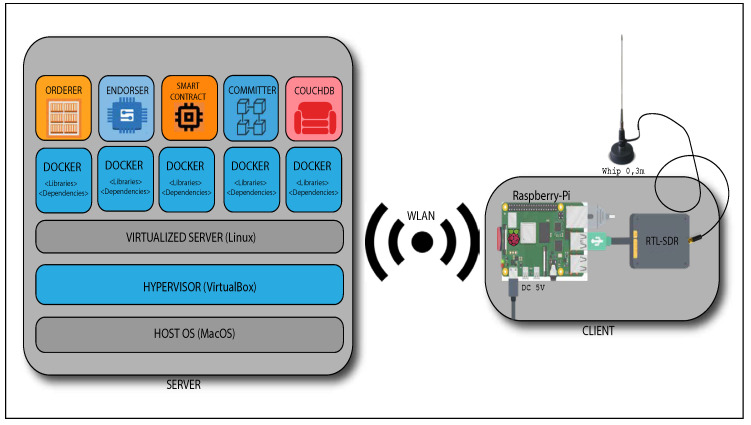
Experiment environment.

Figure 5 presents the experiment environment, where the blockchain client (on the right) is connected to our Linux virtualized server (on the left) and, at the top of it, each blockchain entity running isolated in its container.

To simulate a Naval District blockchain server, we virtualized a Linux server by the VirtualBox Hypervisor [48]. In the Linux server, we virtualized each blockchain peer in Docker’s containers. Each container has its own network interface, enabling its direct communication with the other entities and the sensing nodes. The prototype code and all applications necessary to run the experiment are available in a GitHub public repository [49]. Our virtualized Linux server is set with an Intel Core i5 (1.8 GHz) and 2048 MB of memory. The client’s Raspberry Pi 3 has a Cortex-A53 Quad-core (1.4 GHz) processor and 1048 MB of memory. The blockchain’s server receives the sensing data in an NMEA format through ports 7050 to 7054.

We positioned the AIS VHF antenna near Guanabara Bay (Rio de Janeiro, Brazil) to receive the AIS data of maritime traffic in the area. The AIS data broadcast by the ships are received by the VHF whip antenna and analog-to-digital converted by the SDR dongle. The SDR dongle control program then saves each entry in a text file, which is read by the client application. To simulate an MMS with a vast number of sensing nodes and a large amount of sensing data, we sent 1500 AIS entries, representing 100 nodes sending 15 AIS entries in a minute.

### 5.2. Blockchain Overhead Assessment

**Figure 6:**
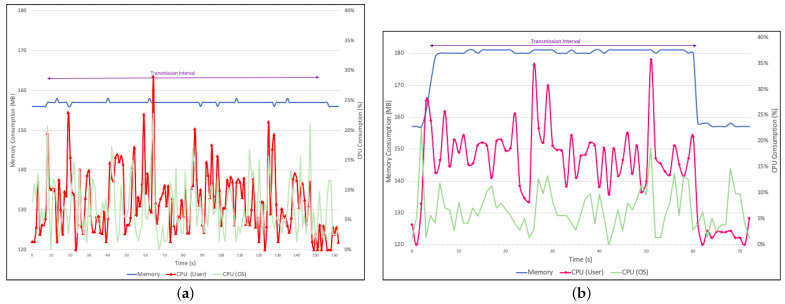
Sensing node sending 1500 AIS entries. (**a**) Sending data via SSH. (**b**) Sending data via blockchain transactions.

First, we set a benchmark using a conventional secure protocol. We compared the client performance by evaluating the CPU and memory usage by sending 1500 AIS entries through the Secure Shell (SSH) protocol and then through the blockchain. We chose SSH due to the fact that the protocol employs both symmetric and asymmetric cryptography. Figure 6a shows the CPU and memory consumption during the process. The red line represents the CPU consumption by the SSH protocol, reaching an average of 8% and a standard deviation of 5%. The light green line represents the CPU consumption by the OS, with an average consumption of 7% during the transmission process. The memory consumption remains stabilized at around 156 MB.

Figure 6b shows the client performance while sending the 1500 AIS entries via blockchain transactions. As expected, we can observe an increase in both CPU and memory consumption. The magenta line represents the blockchain’s client CPU consumption, with an average of 17% and a standard deviation of 8%, however without compromising the OS performance, which remained approximately the same with an average of 6%. Memory consumption stabilized at around 180 MB during transmission.

Our comparison shows a blockchain overhead of 9% in CPU usage and 14.7% in memory usage over the SSH protocol. Nonetheless, the blockchain client uses only 17.6% of all 1024 MB of memory and 16.9% of the CPU in the Raspberry Pi 3. It is also important to highlight that the blockchain transmission was 60% faster, taking about 60 s, while the SSH transmission lasted around 150 s.

The client end is one of the main concerns in our research because these sensing devices are often installed in platforms such as buoys and UAVs, which cannot support heavy payloads. Besides that, these devices need to be prepared for heavy weather conditions and the possibility of tampering by an attack. Hence, these lighter devices do not possess the same computational resources as the system servers. This is why the performance analysis is bold on the sensing nodes.

**Figure 7:**
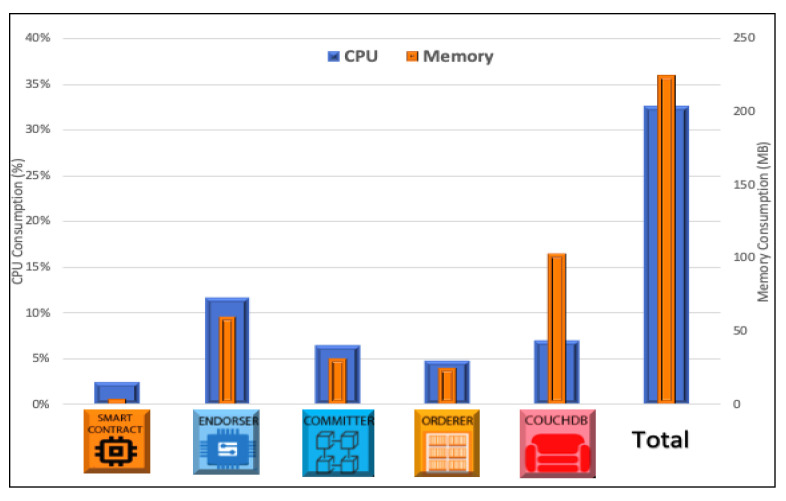
Server performance.

In the server, we analyzed the CPU and memory usage in each blockchain entity, as shown in Figure 7. We evaluated the performance of the smart contract (it runs in a self-container), endorser, committer, orderer, and CouchDB (the local ledger), each one running isolated on its Docker container. The smart contract has a low consumption regarding the the CPU (3%) and memory (4.3 MB) since, in our prototype, it has only the function of processing and verifying the AIS entries. On the other hand, the endorser alone consumed an average of 11.7% of CPU and 102 MB due to its transaction sign function, using asymmetric encryption algorithms.

All containers on our server, however, consumed an average of 224.2 MB, representing 11% of all 2048 MB of the server’s memory and 34.4% of the server’s CPU. As mentioned before, this consumption of computational resources will increase with the blockchain size. Still, Fabric’s blockchain delivers a performance that meets good QoS levels. Moreover, the computational power of a server can be improved more easily to support a bigger network with a larger number of sensing nodes.

### 5.3. Performance with Different Client Hardware Configurations

In order to reduce the scalability problem in terms of client production, we then implemented dockerized blockchain clients. This implementation drastically reduces the time of setting a Raspberry Pi to operate as a blockchain client. In previous works, we faced setbacks with this client preparation due to the Raspberry Pi OS updates and its compatibility with HyperLedger Fabric dependencies.

This client-end modification also allowed us to easily limit the computational resource available to the Docker container operating on the Raspberry Pi. By limiting the hardware available to the container, it is possible to find an optimal point where we can achieve intelligent use of resources, requiring less hardware power, while also consuming less energy. To pinpoint this optimal operation setup, we conducted tests with different limits of the CPU and memory. Note that all CPU percentages specified in this section refer to a percentage of only one core of the Raspberry Pi.

In this second set of experiments, we sent 200 AIS entries and analyzed the client performance with different setups of the CPU and memory available to the client’s container. We then repeated this process 30 times with each setup to reduce the influence of network variables on the results. Initially, we sought the inferior limit of the CPU and memory and found that the blockchain client required at least 18 MB of memory and 10% of the Raspberry Pi’s CPU allocated. Furthermore, the experiments showed that increasing the memory above 30 MB did not significantly reduce the execution time of the client application. For this reason, we did not explore configurations with more than 30 MB of memory.

**Figure 8:**
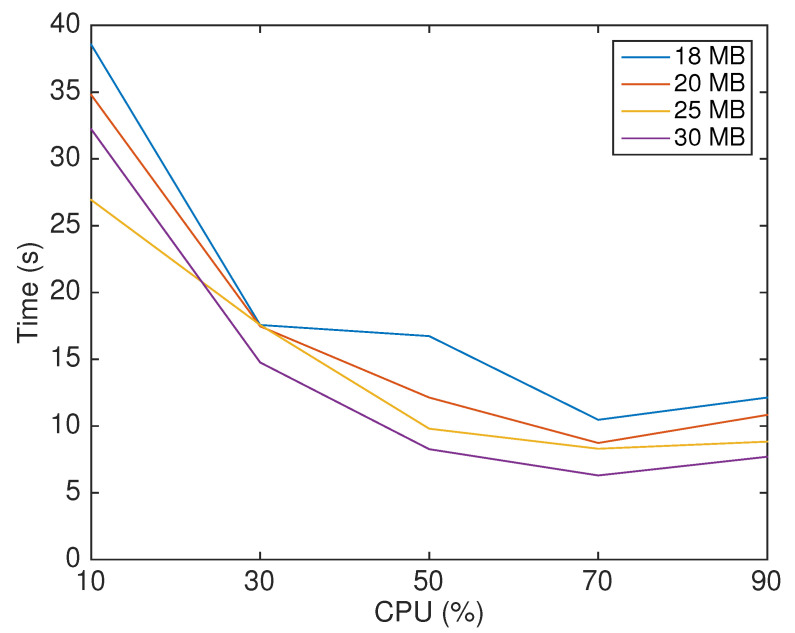
Time performance of the blockchain client for different hardware configurations.

After finding these limits, we focused on finding the optimal setup that required less hardware, and consequently less battery energy, plus delivering the best performance. We tested different hardware configurations, combining the CPU limits of 10%, 30%, 50%, 70%, and 90% and memory limits of 18 MB, 20 MB, 25 MB, and 30 MB. The results presented in Figure 8 show an improvement in performance and an approximately exponential decline of the transmission time for CPU limits from 10% to 70%. However, between 70% and 90% of allocated CPU, we can observe a slight increase in the transmission time. This increase was due to the resource competition between Docker and the OS network calls. Thus, the optimal setup found (considering computational costs and gain in time performance) is using 70% of the CPU of one core of the Raspberry Pi.

**Figure 9:**
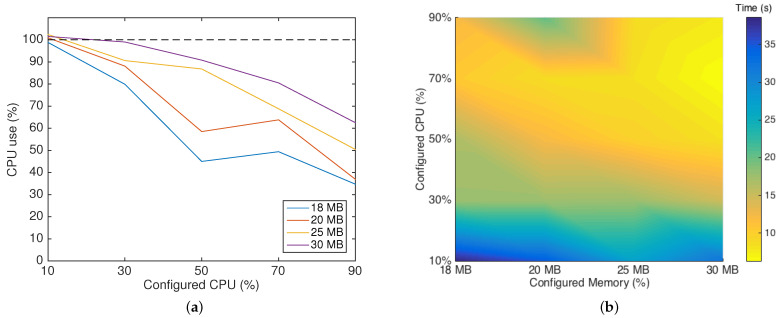
Dockerized client performance. (**a**) Percentage of used CPU in each setup. (**b**) Client performance colormap.

In Figure 9a, we can analyze the percentage of CPU used by the dockerized blockchain client in each configuration. In the 18 MB and 20 MB lines, we can observe an increase in the percentage of CPU usage when we increased the allocated CPU for the container from 50% to 70%. However, the same profile did not occur when the allocated memory was 25 MB and 30 MB. In these cases, the percentage of used CPU steadily declined.

Furthermore, Figure 9b clearly illustrates the best performance area (light yellow), showing that with 70% of the CPU, either the configuration with 25 MB or 30 MB of memory delivered a similar time performance; however, as shown in Figure 9a, with 30 MB, the container used more CPU. Therefore, as the increase in used CPU also implies an increase in energy consumption, we pinpointed 25 MB as the optimal memory size, when aiming at efficiency.

With this dockerized client analysis, we were able to find the optimized hardware for our blockchain-based MMS while addressing some of the scalability problems. Our experiment indicated that it is possible to reduce the resource consumption of the blockchain client, thus requiring less expensive hardware and consuming energy efficiently. Besides, as a nation-scale MMS will demand a great number of sensors, finding the optimal hardware could reduce the costs of the system’s deployment, as well as its maintenance.

We further emphasize that we chose low-cost hardware to demonstrate that blockchain solutions for MMSs are suitable not only for resource-rich projects. Furthermore, the robustness and reliability of blockchain solutions primarily depend on the number of peers that compose the network, demanding more resource redundancy, rather than individual hardware power.

## 6. Conclusions and Future Work

Most naval systems operating currently, including their communication, navigation, and monitoring systems, are poorly mature regarding cybersecurity. Aiming to reduce vulnerabilities in naval systems, this paper presented a blockchain-based MMS model that can leverage security, ensuring sensing data’s integrity, authenticity, and availability.

To fulfill the proposed objectives, we successfully developed a permissioned blockchain prototype on the HyperLedger Fabric platform. We made it available in a public repository to allow other researchers to replicate our experiment. The security analysis demonstrated how the blockchain could help mitigate some MMS vulnerabilities.

We integrated the blockchain prototype with a low-cost AIS receiver developed by the Brazilian Navy and sent 1500 real AIS entries to simulate an MMS operation in a real environment. The experiment allowed us to quantitatively determine the overhead caused by blockchain technology. The results showed that despite the increase in CPU and memory consumption, this overhead is at an acceptable QoS level and is justified by the data security improvements.

Additionally, we presented our dockerized blockchain client implementation to achieve smart resource consumption on the sensing nodes while reducing the scalability problems of a wide-scale IoT network. This experiment allowed us to identify an optimal hardware configuration for the client, thus reducing deployment and maintenance costs and using resources efficiently.

In addition to contributing to the strengthening of MMS cybersecurity and proving the feasibility of using low-cost hardware for blockchain-based AIS receivers, it is important to highlight other implications of this study. The proposed solution and the code provided by this work can be extended to other large-scale maritime sensor systems, including environmental monitoring systems. Furthermore, the methodology herein described for assessing low-cost hardware configurations for blockchain-based clients addresses scalability issues and can be easily replicated in other scopes, such as in wireless sensor networks and IoT technologies. Lastly, this optimized hardware configuration could reduce costs and improve energy efficiency in those systems.

In future work, we plan to continue developing our blockchain prototype to achieve scalability in a full-scale MMS, capable of monitoring the whole coast of a country. In our research, we integrated our prototype with just one sensing system (AIS), and further developments are necessary to extrapolate this integration to different sensing systems in a heterogeneous MMS.

Another subject of further study is the fusing of the blockchain’s decentralization capabilities and AI’s data analysis/decision-making functionality. The symbiosis of these two technologies could allow an MMS to evolve into a command and control system, which could assist naval authorities’ decision-making in the theater of operations. With the high capacity and low latency of blockchain transactions, AI algorithms could be implemented through smart contracts on-chain, building strong processing and data analysis capabilities, which could assist with fast and precise operational insights, while ensuring critical data security requirements.

## Data Availability

Not applicable.

## Abbreviations

The following abbreviations are used in this manuscript:

AWS	Amazon Web Services
AIS	Automatic Identification System
ARPA	Automatic Radar Plotting Aid
CVE	Common Vulnerabilities and Exposures
CFS	Completely Fair Scheduler
CFT	Crash Fault-Tolerant
ECDIS	Electronic Chart Display Information Systems
EEZ	Exclusive Economic Zone
GMDSS	Global Maritime Distress and Safety System
GNSS	Global Navigation Satellite System
IT	Information Technology
INS	Integrated Navigation System
IMO	International Maritime Organization
IoT	Internet of Things
MDA	Maritime Domain Awareness
MMSI	Maritime Mobile Service Identity
MMS	Maritime Monitoring System
MTS	Maritime Transportation System
MSP	Membership Service Provider
MSaaS	Modeling and Simulation-as-a-Service
NMEA	National Marine Electronics Association
NMI	Nautical Mile
ND	Naval District
OS	Operating System
OT	Operational Technology
pAIS	Protected AIS
PoET	Proof of Elapsed Time
PoS	Proof of Stake
PoW	Proof of Work
QoS	Quality of Service
RF	Radio Frequency
SOLAS	Safety of Life at Sea
SSH	Secure Shell
SPF	Single Point of Failure
SDR	Software-Defined Radio
SDK	Software Development Kit
UAV	Unmanned Aerial Vehicles
VTS	Vessel Traffic System
WMAN	Wireless Metropolitan Area Network
